# Smart Antifreeze Hydrogels with Abundant Hydrogen Bonding for Conductive Flexible Sensors

**DOI:** 10.3390/gels8060374

**Published:** 2022-06-13

**Authors:** Bailin Dai, Ting Cui, Yue Xu, Shaoji Wu, Youwei Li, Wu Wang, Sihua Liu, Jianxin Tang, Li Tang

**Affiliations:** National & Local Joint Engineering Research Center for Advanced Packaging Material and Technology, College of Life Sciences and Chemistry, Hunan University of Technology, Zhuzhou 412007, China; daibailin7@163.com (B.D.); cuiting1132@163.com (T.C.); moonxu777@163.com (Y.X.); wushaoji321@163.com (S.W.); 13574763350@163.com (Y.L.); cvery@hut.edu.cn (W.W.); sihua0526@hut.edu.cn (S.L.)

**Keywords:** intelligent gel, antifreeze conductive hydrogel, flexible sensor

## Abstract

Recently, flexible sensors based on conductive hydrogels have been widely used in human health monitoring, human movement detection and soft robotics due to their excellent flexibility, high water content, good biocompatibility. However, traditional conductive hydrogels tend to freeze and lose their flexibility at low temperature, which greatly limits their application in a low temperature environment. Herein, according to the mechanism that multi−hydrogen bonds can inhibit ice crystal formation by forming hydrogen bonds with water molecules, we used butanediol (BD) and N−hydroxyethyl acrylamide (HEAA) monomer with a multi−hydrogen bond structure to construct LiCl/p(HEAA−*co*−BD) conductive hydrogel with antifreeze property. The results indicated that the prepared LiCl/p(HEAA−*co*−BD) conductive hydrogel showed excellent antifreeze property with a low freeze point of −85.6 °C. Therefore, even at −40 °C, the hydrogel can still stretch up to 400% with a tensile stress of ~450 KPa. Moreover, the hydrogel exhibited repeatable adhesion property (~30 KPa), which was attributed to the existence of multiple hydrogen bonds. Furthermore, a simple flexible sensor was fabricated by using LiCl/p(HEAA−*co*−BD) conductive hydrogel to detect compression and stretching responses. The sensor had excellent sensitivity and could monitor human body movement.

## 1. Introduction

Hydrogel is a kind of soft−wet material with a three−dimensional polymer network [1,2,3]. In recent years, hydrogels have been widely applied in the fields of intelligent sensing [4,5], biomedicine [6,7,8,9,10], and human health monitoring [11,12,13] due to its high stretchability, ionic conductive path, and high water content [14,15,16]. Electronic or ionic conductivity is a necessary condition to ensure that hydrogel can transform external mechanical stimulation into easily collected electrical signals. However, most hydrogels lack conductivity due to the insulating properties of conventional polymer networks. Commonly used methods to enhance the conductivity of hydrogel include filling conductive fillers (graphene [17,18], carbon nanotubes [19,20], polypyrrole [21,22] and polyaniline [23], etc.) or adding soluble electrolytes (e.g., LiCl, NaCl, and (NH_4_)_2_SO_4_) [24,25,26,27,28]. Although the introduction of conductive fillers provides ionic or electronic conductivity for hydrogels, it also raises some issues, such as difficult dispersion of conductive nanomaterials and easy agglomeration, leading to the loss of mechanical properties and electrical conductivity of hydrogels. In addition, the inherent incompatibility between a rigid conductive polymer network and flexible networks results in a decrease in the flexibility of hydrogels. On the other hand, the high water content of hydrogel makes it easy to freeze at low temperature, losing its flexibility and conductivity. Therefore, how to endow hydrogels with conductivity and improve their frost resistance remains a huge challenge.

The introduction of a soluble electrolyte (e.g., LiCl, NaCl, and (NH_4_)_2_SO_4_) is one of the most commonly used methods, which can not only improve the conductive ability of hydrogels, but also inhibit the generation of ice crystals, and reduce the freezing point of hydrogels [29,30,31]. Zhang and co−workers prepared anti−freezing hydrogels by soaking hydrogel in (NH_4_)_2_SO_4_ solution to replace the water of the hydrogel. The resultant hydrogel showed excellent ionic conductivity (~2.7 S/m) at −40 °C, and maintained good flexibility in a wide temperature range (−40~25 °C) [32]. Nevertheless, too much salt in a hydrogel will lead to undesirable mechanical properties, which is not conducive to practical applications of hydrogels. Another way to endow hydrogel with frost resistance is to introduce an organic solvent, which reduces the freezing point of hydrogels through the interaction of organic solvents with water molecules. Based on this strategy, Wu et al. designed a gelatin/N−hydroxyethyl acrylamide/glycerol/lithium chloride double network hydrogel by one pot−heating−cooling method [33]. The prepared hydrogel possesses excellent mechanical property (tensile stress/strain of 2.14 MPa/1637.49%). Moreover, due to the introduction of glycerol, the hydrogel shows good anti−freezing ability. It still has good flexibility at −40 °C and can light LED at −80 °C. However, the introduction of an organic solvent with a low dielectric coefficient reduces the ionic moving speed of a hydrogel, resulting in low conductivity the hydrogel in question.

Herein, we designed a LiCl/p(HEAA−*co*−BD) conductive hydrogel with anti−freezing ability and a high mechanical property. BD is a monomer with polyol structure, which is similar to ethylene glycol and glycerol. The polyol structure of BD is easy to combine with free water through hydrogen bonding, thus inhibiting the formation of ice crystals and reducing the freezing temperature of hydrogel. Moreover, HEAA was selected due to its rich amide and hydroxyl groups, which can form more hydrogen bonds between inter− and intra− polymer chains, thus enhancing the mechanical properties and adhesion properties of hydrogel. Therefore, the obtained LiCl/p(HEAA−*co*−BD) conductive hydrogel has a low freeze point at −85.6 °C, and the tensile stress/strain of the hydrogel can reach 450 KPa/400% at −40 °C. In addition, hydrogel shows excellent adhesive ability (~150 KPa) on various non−porous substrates, such as rubber, plastic, foam, paper and other substrates. Furthermore, a flexible sensor based on a LiCl/p(HEAA−*co*−BD) conductive hydrogel was prepared, which can effectively respond to different pressure and monitor human limb bending. This design strategy of a low−temperature conductive hydrogel will effectively expand the application of hydrogel.

## 2. Results and Discussion

### 2.1. Preparation of LiCl/p(HEAA−co−BD) Antifreeze Conductive Hydrogel

**Figure 1:**
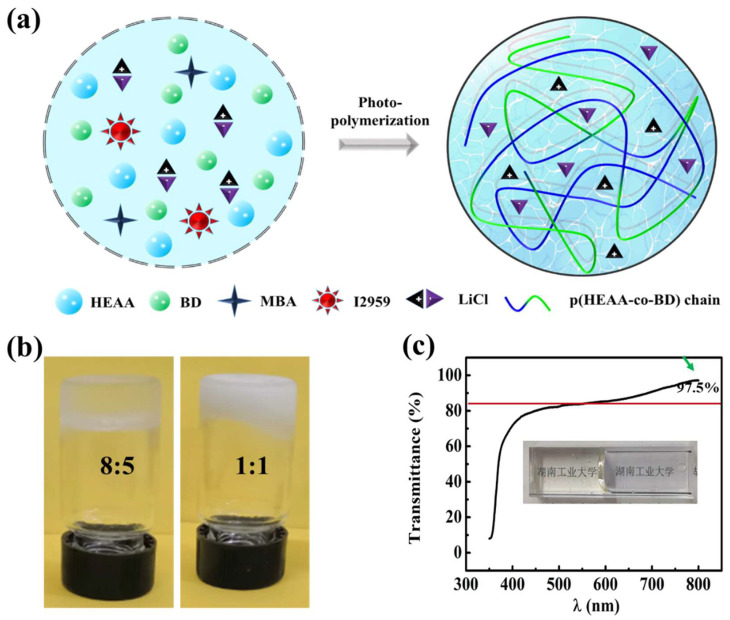
(**a**) Schematics of preparation of LiCl/p(HEAA−*co*−BD) antifreeze conductive hydrogel; (**b**) Frost resistance of HEAA hydrogels with different concentrations of HEAA at −20 °C; (**c**) The transmittance of LiCl/p(HEAA−co−BD) antifreeze conductive hydrogel in a colorimetric dish.

As shown in Figure 1a, LiCl/p(HEAA−*co*−BD) antifreeze conductive hydrogels were synthesized by a photopolymerization method (Figure 1). The specific synthesis steps are as follows: BD, HEAA, MBA, LiCl and I2959 were added to a bottle with water. After that, the prepared solution was injected into the mold through a syringe and placed under 8 W UV light for 1 h. In this process, free radicals were generated by the decomposition of I2959 to induce the copolymerization of BD and HEAA, which further formed a three−dimensional polymer network by cross−linking agent MBA. Among them, LiCl is uniformly dispersed in the polymer hydrogel network, which gives the hydrogel excellent electrical conductivity by forming movable free hydration ions. In addition, the hydrogel exhibited excellent freezing resistance by inhibiting the formation of ice crystals in hydrogels through the multiple hydrogen bonding of butanediol and N−hydroxyethyl acrylamide with H_2_O at low temperature. As shown in Figure 1b, a pure pHEAA hydrogel (m_HEAA_:m_H2O_ = 1:1) was frozen at −20 °C, showing poor frost resistance. The formed pure pHEAA hydrogel did not freeze at −20 °C when HEAA concentration was increased to 61.5 wt% (m_HEAA_:m_H2O_ = 8:5). Obviously, with the increase in HEAA concentration, the content of hydrophilic functional groups in the pHEAA polymer chain increased, and the number of hydrogen bonds formed with free water increased, thus enhancing the freezing resistance of the polymer.

On the side, the transparency of the LiCl/p(HEAA−*co*−BD) anti−freezing conductive hydrogel was measured with a UV−vis spectrometer. As shown in Figure 1c, the transmission of the hydrogel was as high as 80–90% in the wavelength range of 400–700 nm, which was close to the transmission rate of some commercial transparent films. In addition, the Chinese characters (Chinese name of Hunan University of Technology) printed on the paper were clearly observed through the cuvette containing the hydrogel, and the characters of “Hunan University of Technology” were clearly observed. Compared with the blank next to them, there was no obvious blurring of the characters, showing high permeability.

### 2.2. Comparison of Anti−Freezing Capacity of Different Hydrogel Components

The anti−freezing ability of different hydrogel components was shown in Table 1. All hydrogel prepolymerized solutions were transparent. Then, the prepolymerized solution was polymerized by UV light to form a transparent hydrogel at room temperature (RT). The anti−freezing ability of the hydrogel was judged by the change of its color at −20 °C. The hydrogel still showed high light transmittance after being frozen at −20 °C for 12 h, which indicated that the hydrogel did not freeze at −20 °C. When this series of hydrogels were further frozen at −80 °C for 12 h, it was found that the LiCl/p(HEAA−*co*−BD)/H_2_O hydrogel was still transparent, which means that there was no freezing in this hydrogel, while all or part of the LiCl/pHEAA/H_2_O, p(HEAA−*co*−BD)/H_2_O and pHEAA/H_2_O hydrogels were milky white. This means that the LiCl/p(HEAA−*co*−BD)/H_2_O hydrogel had the best anti−freezing ability, which was attributed to the interaction between the monomer and LiCl with water molecules.

### 2.3. Low−Temperature Tensile Properties of LiCl/p(HEAA−co−BD) Anti−Freezing Conductive Hydrogels

**Figure 2:**
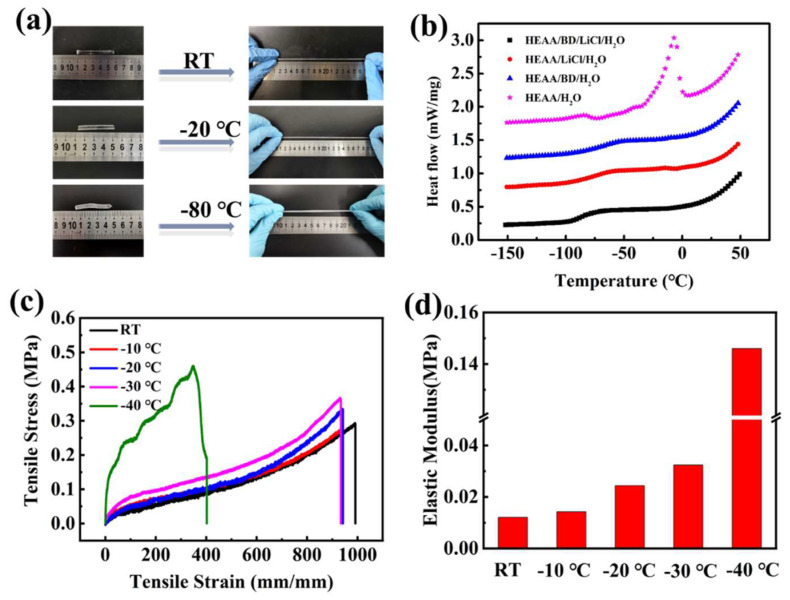
(**a**) LiCl/p(HEAA−*co*−BD) hydrogel stretched under RT, −20 °C and −80 °C; (**b**) DSC curves of hydrogels with different components; (**c**) Stress–strain curves and; (**d**) Elastic modulus of LiCl/p(HEAA−*co*−BD) hydrogel at RT, −10 °C, −20 °C, −30 °C, and −40 °C, respectively.

Tensile properties of the LiCl/p(HEAA−*co*−BD) hydrogel at different temperatures were shown in Figure 2a. At RT, −20 °C and −80 °C, the hydrogel shows good tensile properties and excellent flexibility.

In order to quantitatively test and compare the anti−freezing ability of this series of hydrogels, we presented the DSC curves of hydrogels (Figure 2b). The glass transition temperatures of the LiCl/pHEAA/H_2_O, p(HEAA−*co*−BD)/H_2_O and pHEAA/H_2_O hydrogels were −51.8 °C, −64.9 °C and −17.1 °C, respectively. The above data demonstrated that both BD and LiCl have excellent anti−freezing ability. It can be clearly observed that after combining BD and LiCl with pHEAA/H_2_O hydrogel, the resultant LiCl/p(HEAA−*co*−BD)/H_2_O hydrogel had the lowest glass transition temperature (Tg) of −85.6 °C. This method overcomes the limitation that traditional antifreeze hydrogels are mostly based on organic hydrogels and obtains excellent antifreeze water−based hydrogels.

The tensile properties of the hydrogel were quantitatively characterized by a tensile test at a low temperature from RT to −40 °C (Figure 2c). The hydrogel showed superb tensile properties, which can be stretched up to 200 KPa/1000% at RT. When the temperature was gradually reduced to −10 °C, −20 °C and −30 °C, the fracture strain of the hydrogel did not change significantly, but the fracture stress gradually increased. When the temperature was reduced to −40 °C, the fracture strain shrank to ~400% and the fracture stress reached a maximum of 450 KPa. Obviously, when the temperature decreases from RT to −40 °C, the elastic modulus of the hydrogel showed a similar trend to the tensile properties of the hydrogel (Figure 2d). This occurs as, when the temperature drops, the hydrogel gradually begins to change from a highly elastic state to a glassy state, where it becomes harder and less flexible.

### 2.4. Adhesion of LiCl/p(HEAA−co−BD) Hydrogel

**Figure 3:**
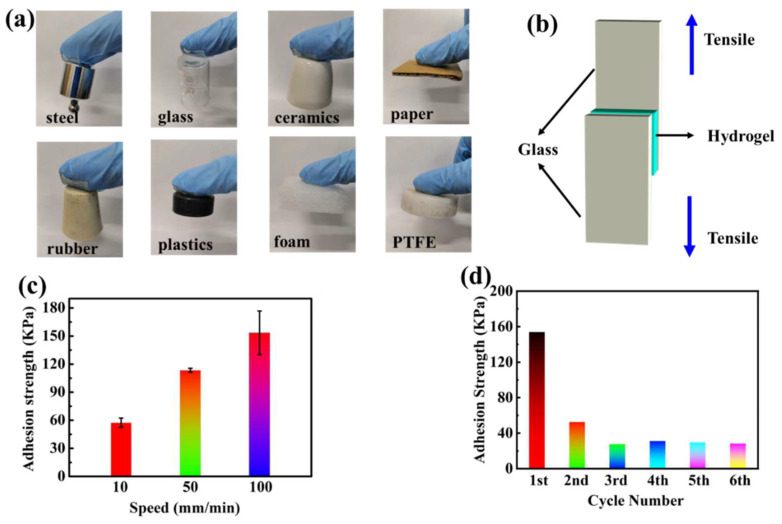
(**a**) LiCl/p(HEAA−*co*−BD) hydrogels adhere to different substrates; (**b**) Schematic diagram of lap shear testing for strip strength; (**c**) Effect of peel speed on adhesion strength of hydrogels; (**d**) multiple adhesion of hydrogels.

The LiCl/p(HEAA−*co*−BD) hydrogel possesses excellent adhesion due to it contains a large number of hydrogen bonds. First, the adhesive properties of hydrogel on various non−porous substrates were qualitatively tested. As shown in Figure 3a, the LiCl/p(HEAA−*co*−BD) hydrogel not only could adhere to inorganic materials such as stainless steel, ceramics and glass, but also can adhere to organic materials such as rubber, plastic, foam and cardboard. In addition to showing adhesion to hydrophilic substances, the hydrogel also adhered to hydrophobic PTFE substrates, overcoming the problem that most adhesives do not adhere to hydrophobic materials. Furthermore, the adhesion strength of the hydrogel on glass substrates was quantitatively tested. Figure 3b shown a schematic diagram of 180° peeling test for exploring the adhesion of hydrogel [34]. The adhesion strength of the LiCl/p(HEAA−*co*−BD) hydrogel at different peeling speeds is shown in Figure 3c. It can be seen that the adhesion strength of the hydrogel gradually increased from ~60 KPa to 150 KPa as the peeling speed increased from 10 mm/min to 100 mm/min, showing obvious speed−dependent properties.

More than that, the LiCl/p(HEAA−*co*−BD) hydrogel demonstrated excellent reversible adhesion properties. As shown in Figure 3d, the reversible adhesive strength of the hydrogel was tested five times with a resting time of two minutes during the two peeling tests. The results showed that the hydrogel adhesion strength decreased rapidly from ~160 KPa to ~50 KPa when the second adhesion test was performed. This occurs as, in the early stage of synthesis, the prepolymerized solution could be filled into the micropores on the glass substrate, which can eliminate some invisible bubbles on the glass substrate. After an hour photopolymerization, the hydrogel fragments in the micropores form a whole with the surface hydrogel, so the strength of hydrogel is higher than cyclic adhesion. After the first peeling, some hydrogel fragments would remain on the surface of the hydrogel, and when re−adhering, these hydrogel fragments would contact with the bulk hydrogel to form bubbles, thus reducing the adhesion ability when re−adhering. Due to the above reasons, the second adhesive strength will be lower than the first adhesive strength. When the peeling−adhesion test was repeated several times, the adhesion strength was basically stabilized at ~30 KPa. This is due to the immediate recovery of dynamic reversible interaction. Therefore, the LiCl/p(HEAA−*co*−BD) hydrogel showed relatively weak and relatively stable adhesive strength in a limited recovery time.

### 2.5. Conductivity of LiCl/p(HEAA−co−BD) Antifreeze Hydrogels

**Figure 4:**
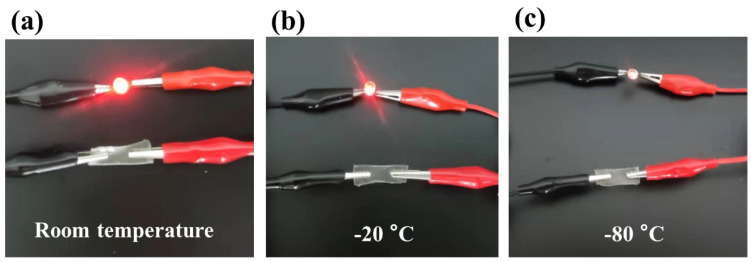
Conductivity of LiCl/p(HEAA−*co*−BD) hydrogel at different temperatures: (**a**) RT; (**b**) −20 °C and; (**c**) −80 °C.

The conductivity of the LiCl/p(HEAA−*co*−BD) hydrogel was tested by serially connecting it into the circuit of a battery with 8V at RT, −20 °C and −80 °C, respectively. The results were shown in Figure 4. The hydrogel not only had excellent conductivity at RT (LED emitted bright light), but also possessed conductivity at a low temperature (LED can be lit). Among them, since LiCl can be hydrogenated to freely mobile Li^+^ and Cl^−^ at RT, which can allow the hydrogel to exhibit conductive ability, the LED in the path exhibits bright emission. With the decrease in temperature, the decline in mobility of hydrated Li^+^, resulting in the decrease in the conductivity of hydrogel.

### 2.6. Sensing Performance of LiCl/p(HEAA−co−BD) Hydrogel

**Figure 5:**
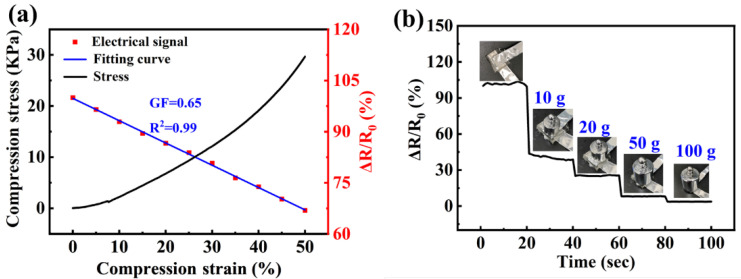
(**a**) Compression stress and compression strain and (**b**) Resistance changes of hydrogel response to different weights.

Figure 5a shows the resistance changes of LiCl/p(HEAA−*co*−BD) hydrogel with compressive strain. It could be seen that the resistance changes of hydrogel showed an obvious linear relationship (R^2^ = 0.99) and GF = 0.65 with the compressive strain of 0–50%. Furthermore, we tested the pressure response properties of the hydrogel by adding heavy weights. As shown in Figure 5b. As the mass of the weight applied to the hydrogel increases from zero to 100 g, the resistance changes rate of hydrogel decreased from 100% to 4%, showing excellent pressure response properties.

**Figure 6:**
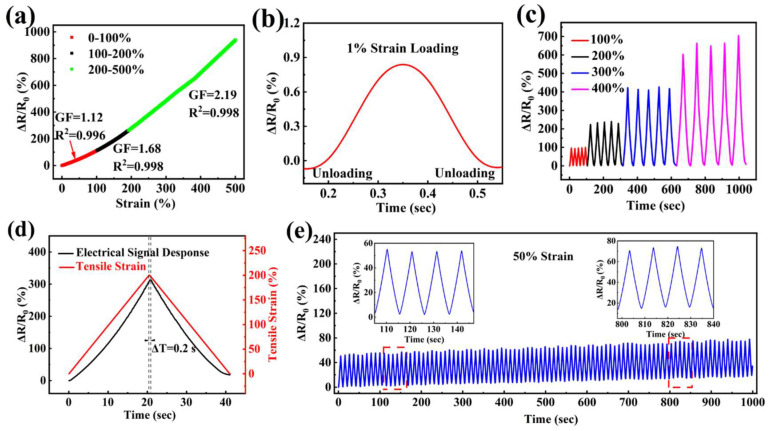
(**a**) GF of the LiCl/p(HEAA−co−BD) hydrogel flexible sensor. The change of relative resistance of the hydrogel flexible sensor during the loading–unloading cycle with a strain of (**b**) 1%, (**c**) 100–400%. (**d**) time−resolved responses of hydrogel flexible sensor. (**e**) The stability of the hydrogel flexible sensor.

The sensitivity of the strain sensor, based on the LiCl/p(HEAA−*co*−BD) hydrogel, was also assessed. The GF were 1.12, 1.68 and 2.19 in the tensile strain range of 0–100%, 100–200% and 200–500% (Figure 6a), respectively, which exhibited excellent sensitivity. Particularly, the obvious signals were successfully gained in the process of loading–unloading under strain of 1%, 100–400%, respectively. (Figure 6b,c). Additionally, the response time of the hydrogel flexible sensor was simultaneously recorded at the stretching speed of 100 mm/min and 200% strain (Figure 6d). The results showed that the response time was only 0.2 s, and it is proved that the hydrogel flexible sensor could immediately respond to the changes in external strain. The stability of the flexible sensor was tested via sixty times loading–unloading cycles with 50% strain. As shown in Figure 6e, the relative resistance of sensor has remained almost unchanged during the cyclic deformations (Figure 6e inset). The deviation of relative resistance was less than 1%, which means that the flexible sensor based on the LiCl/p(HEAA−*co*−BD) hydrogel shows high stability.

**Figure 7:**
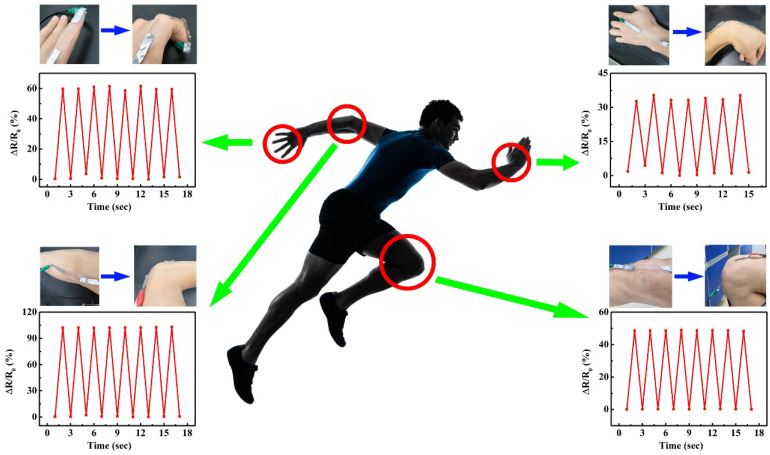
Applications of the LiCl/p(HEAA−*co*−BD) hydrogel for monitoring human motion.

In view of the high tensile property, good electrical conductivity, adhesive property and strain response capability of this anti−freezing hydrogel. A flexible wearable sensor for monitoring human motion was prepared based on the LiCl/p(HEAA−*co*−BD) hydrogel. As shown in Figure 7, the hydrogel based flexible sensor was directly adhered to each joint of the human body. Then, the human movement signals were collected by an electrochemical workstation. The results showed that the relative resistance of hydrogel varied from 0 to 60% with the stretching and bending of a finger, and when the wrist, elbow, and knee were straight and bent, the relative resistance changes of the hydrogel were 30%, 100%, and 50%, respectively. Since the relative deformability of fingers and elbows is greater than that of wrists and knees, the relative resistance of hydrogels changes more obviously with bending deformation.

## 3. Conclusions

In conclusion, LiCl/p(HEAA−*co*−BD) hydrogels with freeze resistance and electrical conductivity were prepared by one−pot photopolymerization with different antifreeze components, such as LiCl, BD and HEAA. Due to the interaction between the multi−hydrogen bonding molecular network with water molecules and the hydration of LiCl, the hydrogel showed a low freezing point of −85.8 °C. Moreover, the hydrogel exhibited excellent tensile strength (the tensile stress/strain of the hydrogel could reach 450 KPa/400% at −40 °C) and conductivity (the LED could still be lit at −80 °C) at low temperature. In addition, based on the dynamic reversible property of a hydrogen bond, the hydrogel also showed a reversible adhesion strength of ~30 KPa. Finally, the flexible sensor based on a LiCl/p(HEAA−*co*−BD) hydrogel was prepared, which showed a high sensitivity (GF = 2.19) and a fast response speed (0.2 s) to external force. Based on the above advantages, the hydrogel can effectively monitor the movements of human limbs (such as finger bending, knee bending, wrist bending and elbow bending).

## 4. Experimental Parts

Experimental reagents and specifications. N−hydroxyethyl acrylamide (HEAA, 98%, chemically pure) was purchased from TCL Reagents Inc. 2−Hydroxy−4′−(2−hydroxyethoxy)−2−methylpropiophenone (I2959, 99%, chemically pure) and N,N’−methylene bisacrylamide (MBA, 99%, chemically pure) were purchased from Shanghai Aladdin Reagent Co. Butenediol (BD, 98%, chemically pure) was purchased from Jiangsu Aikang Biopharmaceutical R&D Co. Lithium chloride (LiCl, analytical purity) was purchased from Sinopharm Reagent Co. Deionized water was prepared by Milli−Q system water purification system, with an impedance of 18.2 MΩ/cm. All reagents were not further purified before use.

Preparation of LiCl/p(HEAA−*co*−BD) antifreeze conductive hydrogel. LiCl/P(HEAA−co−BD) conductive hydrogels with anti−freezing ability was prepared by photo−initiated radical polymerization reaction. The specific synthesis steps are as follows: First of all, the reactants of LiCl (0.21 g, 3 wt.%), HEAA (3.5 g, 50 wt.%), BD (0.7 g, 10 wt.%), MBA (6.8 mg), and photo−initiator I2959 (68 mg) were added to a glass bottle containing deionized water (2.8 g, 40 wt.%). Then, the mixture solution was stirred to form a clear solution. The solution was injected into a glass mold with a thickness of 1 mm through a syringe, and then transferred to an 8 W 365 nm UV light for polymerization for 1 h. After polymerization, the transparent hydrogel was formed. In order to facilitate the removal of the prepared hydrogel from the mold, a layer of PET film was covered on the contact surface between the glass and the hydrogel.

Mechanical property test. Tensile tests were carried out on a universal tester (AGS−X) equipped with 1000 N load cell at a speed of 100 mm/min. The hydrogel specimen was cut into a dumbbell shape with an effective stretching length of 25 mm, a width of 4 mm and a thickness of 1 mm, and mounted on the chuck of the stretching machine. Stretched at a rate of 100 mm/min until the hydrogel fractured. The tensile strain (*ε*) was calculated by the following equation:

*ε* = *l_t_/l*_0_ (1)

where *l*_0_ is the original length of the hydrogel and *l_t_* is the length of the hydrogel after stretching. The tensile stress (*σ*) is the tensile force applied per unit area, and the formula is as follows:

*σ* = *F/A*_0_ (2)

where *F* is the tensile force and *A*_0_ is the cross−sectional area of the hydrogel sample.

Low temperature tensile test. The tensile stress and tensile strain of hydrogels below zero temperature were tested by a universal tester (Zwick Roell Z010) with an environmental chamber. Before stretching, each sample was kept at the test temperature for 20 min to ensure that the temperature of the sample was the same as that of the external environment. The stretching rate was also controlled at 100 mm/min.

Differential Scanning Calorimetry (DSC). DSC test was performed using NETZSCH DSC 200F3 at a heating rate of 10 °C/min in a set temperature range under nitrogen protection.

Adhesion strength test. The adhesive strength of the hydrogels was determined using a 180° peeling test at a peeling−set speed. The hydrogels were prepared into cuboids (length, 10 mm; width, 10 mm; thickness, 2 mm). Adhesive strength (*g*) was calculated as follows:

*g* = *F_max_/S* (3)

where *F_max_* is the maximum force applied in the stripping process, and *S* is the area of the hydrogel sample.

Transmittance test. The UV−vis absorption spectrum of the hydrogel was acquired using a UV−vis spectrophotometer (TU−1810) at a wavelength scanning range from 400 to 800 nm and a scanning rate of 100 nm/min.

Sensing performance test. Compression test electrochemical performance test: Compression response of conductive gels to different masses of weights: The LiCl/p(HEAA−*co*−BD) conductive hydrogel was made into a cylinder with a diameter of 1 cm and a height of 1 cm, and each of the two cross sections was connected to the electrochemical workstation through an aluminum sheet. The gage factor (GF) is calculated according to the following formula:

GF = △*R/R*_0_ × 100% (4)

where *R*_0_ is the original resistance, and △*R* is the change of resistance during stretching.

## Figures and Tables

**Table 1:** Freezing resistance of each component hydrogel.

	LiCl/p(HEAA−co−BD)/H_2_O	LiCl/pHEAA/H_2_O	LiCl/pHEAA/H_2_O	pHEAA/H_2_O
Monomeric ratios (g:g)	0.45:8:2:5	0.45:8:5	8:2:5	8:5
Pre−polymerized liquid	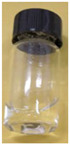	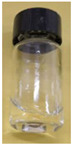	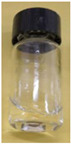	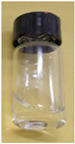
Post−aggregation	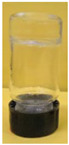	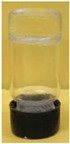	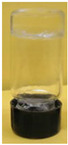	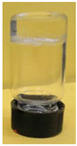
−20 ℃	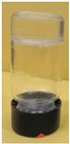	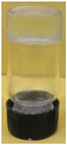	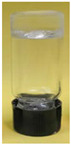	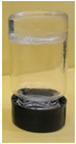
−80 ℃	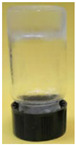	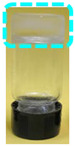	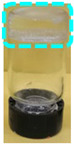	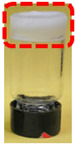
Glass transition temperature	−85.6 °C	−51.8 °C	−64.9 °C	−17.1 °C

## Data Availability

The data presented in this study are available on request from the corresponding author.

## References

[1] Tang J., Huang J., Zhou G., Liu S. (2020). Versatile fabrication of ordered cellular structures double network composite hydrogel and application for cadmium removal. J. Chem. Thermodyn..

[2] Wu Z., Yang X., Wu J. (2021). Conductive hydrogel−and organohydrogel−based stretchable sensors. ACS Appl. Mater. Interfaces.

[3] Zhang D., Tang Y., Zhang Y., Yang F., Liu Y., Wang X., Yang J., Gong X., Zheng J. (2020). Highly stretchable, self−adhesive, biocompatible, conductive hydrogels as fully polymeric strain sensors. J. Mater. Chem. A.

[4] Yang C., Su F., Xu Y., Ma Y., Tang L., Zhou N., Liang E., Wang G., Tang J. (2022). pH Oscillator−Driven Jellyfish−like Hydrogel Actuator with Dissipative Synergy between Deformation and Fluorescence Color Change. ACS Macro Lett..

[5] Tang L., Zhang D., Gong L., Zhang Y., Xie S., Ren B., Liu Y., Yang F., Zhou G., Chang Y. (2019). Double−network physical cross−linking strategy to promote bulk mechanical and surface adhesive properties of hydrogels. Macromolecules.

[6] Chakraborty P., Oved H., Bychenko D., Yao Y., Tang Y., Zilberzwige-Tal S., Wei G., Dvir T., Gazit E. (2021). Nanoengineered Peptide-Based Antimicrobial Conductive Supramolecular Biomaterial for Cardiac Tissue Engineering. Adv. Mater..

[7] Liu K., Wei S., Song L., Liu H., Wang T. (2020). Conductive hydrogels—A novel material: Recent advances and future perspectives. J. Agric. Food Chem..

[8] Zhao Y., Zhu Z.S., Guan J., Wu S.J. (2021). Processing, mechanical properties and bio−applications of silk fibroin−based high−strength hydrogels. Acta Biomater..

[9] Dong Y., Zhuang H., Hao Y., Zhang L., Yang Q., Liu Y., Qi C., Wang S. (2020). Poly (N−isopropyl−acrylamide)/poly (γ−glutamic acid) thermo−sensitive hydrogels loaded with superoxide dismutase for wound dressing application. Int. J. Nanomed..

[10] Rowland M.J., Parkins C.C., McAbee J.H., Kolb A.K., Hein R., Loh X.J., Watts C., Scherman O.A. (2018). An adherent tissue−inspired hydrogel delivery vehicle utilised in primary human glioma models. Biomaterials.

[11] Xu L., Chen Y., Guo Z., Tang Z., Luo Y., Xie S., Li N., Xu J. (2021). Flexible Li^+^/agar/pHEAA double−network conductive hydrogels with self−adhesive and self−repairing properties as strain sensors for human motion monitoring. React. Funct. Polym..

[12] Darabi M.A., Khosrozadeh A., Mbeleck R., Liu Y., Chang Q., Jiang J., Cai J., Wang Q., Luo G., Xing M. (2017). Skin-inspired multifunctional autonomic-intrinsic conductive self-healing hydrogels with pressure sensitivity, stretchability, and 3D printability. Adv. Mater..

[13] Lu J., Han X., Dai L., Li C., Wang J., Zhong Y., Yu F., Si C. (2020). Conductive cellulose nanofibrils−reinforced hydrogels with synergetic strength, toughness, self−adhesion, flexibility and adjustable strain responsiveness. Carbohydr. Polym..

[14] Wu S., Tang L., Xu Y., Yao J., Tang G., Dai B., Wang W., Tang J., Gong L. (2022). A self−powered flexible sensing system based on a super−tough, high ionic conductivity supercapacitor and a rapid self−recovering fully physically crosslinked double network hydrogel. J. Mater. Chem. C.

[15] Tang L., Wu S., Xu Y., Li Y., Dai B., Yang C., Liu A., Tang J., Gong L. (2022). Design of a DNA-Based Double Network Hydrogel for Electronic Skin Applications. Adv. Mater. Technol..

[16] Tang L., Wu S., Qu J., Gong L., Tang J. (2020). A review of conductive hydrogel used in flexible strain sensor. Materials.

[17] He Q., Liu J., Liu X., Li G., Deng P., Liang J. (2018). Manganese dioxide Nanorods/electrochemically reduced graphene oxide nanocomposites modified electrodes for cost−effective and ultrasensitive detection of Amaranth. Colloids Surf. B Biointerfaces.

[18] Alam A., Meng Q., Shi G., Arabi S., Ma J., Zhao N., Kuan H.C. (2016). Electrically conductive, mechanically robust, pH−sensitive graphene/polymer composite hydrogels. Compos. Sci. Technol..

[19] Xiang K., Wen X., Hu J., Wang S., Chen H. (2019). Rational Fabrication of Nitrogen and Sulfur Codoped Carbon Nanotubes/MoS2 for High-Performance Lithium–Sulfur Batteries. ChemSusChem.

[20] Hsiao L.-Y., Jing L., Li K., Yang H., Li Y., Chen P.-Y. (2020). Carbon nanotube−integrated conductive hydrogels as multifunctional robotic skin. Carbon.

[21] Sun M., Wu X., Liu C., Xie Z., Deng X., Zhang W., Huang Q., Huang B. (2018). The In Situ grown of activated Fe−N−C nanofibers derived from polypyrrole on carbon paper and its electro−catalytic activity for oxygen reduction reaction. J. Solid State Electrochem..

[22] Zhao L., Li X., Li Y., Wang X., Yang W., Ren J. (2021). Polypyrrole−Doped Conductive Self−Healing Composite Hydrogels with High Toughness and Stretchability. Biomacromolecules.

[23] Wu C., Shen L., Lu Y., Hu C., Liang Z., Long L., Ning N., Chen J., Guo Y., Yang Z. (2021). Intrinsic Antibacterial and Conductive Hydrogels Based on the Distinct Bactericidal Effect of Polyaniline for Infected Chronic Wound Healing. ACS Appl. Mater. Interfaces.

[24] Tang L., Gong L., Xu Y., Wu S., Wang W., Zheng B., Tang Y., Zhang D., Tang J., Zheng J. (2022). Mechanically Strong Metal–Organic Framework Nanoparticle−Based Double Network Hydrogels for Fluorescence Imaging. ACS Appl. Nano Mater..

[25] Xu H., Lv Y., Qiu D., Zhou Y., Zeng H., Chu Y. (2019). An ultra−stretchable, highly sensitive and biocompatible capacitive strain sensor from an ionic nanocomposite for on−skin monitoring. Nanoscale.

[26] Kang T.H., Chang H., Choi D., Kim S., Moon J., Lim J.A., Lee K.Y., Yi H. (2019). Hydrogel−templated transfer−printing of conductive nanonetworks for wearable sensors on topographic flexible substrates. Nano Lett..

[27] Peng H., Xin Y., Xu J., Liu H., Zhang J. (2019). Ultra−stretchable hydrogels with reactive liquid metals as asymmetric force−sensors. Mater. Horiz..

[28] Zhang Q., Liu X., Duan L., Gao G. (2019). Ultra−stretchable wearable strain sensors based on skin−inspired adhesive, tough and conductive hydrogels. Chem. Eng. J..

[29] Sui X., Guo H., Cai C., Li Q., Wen C., Zhang X., Wang X., Yang J., Zhang L. (2021). Ionic conductive hydrogels with long−lasting antifreezing, water retention and self−regeneration abilities. Chem. Eng. J..

[30] Kong W., Wang C., Jia C., Kuang Y., Pastel G., Chen C., Chen G., He S., Huang H., Zhang J.J.A.M. (2018). Muscle-Inspired Highly Anisotropic, Strong, Ion-Conductive Hydrogels. Adv. Mater..

[31] Liu Y., Wang W., Gu K., Yao J., Shao Z., Chen X. (2021). Poly (vinyl alcohol) Hydrogels with Integrated Toughness, Conductivity, and Freezing Tolerance Based on Ionic Liquid/Water Binary Solvent Systems. ACS Appl. Mater. Interfaces.

[32] Sui X., Guo H., Chen P., Zhu Y., Wen C., Gao Y., Yang J., Zhang X., Zhang L. (2020). Zwitterionic osmolyte-based hydrogels with antifreezing property, high conductivity, and stable flexibility at subzero temperature. Adv. Funct. Mater..

[33] Tang L., Wu S., Xu Y., Cui T., Li Y., Wang W., Gong L., Tang J. (2021). High toughness fully physical cross−linked double network organohydrogels for strain sensors with anti−freezing and anti−fatigue properties. Mater. Adv..

[34] Jing X., Mi H.Y., Lin Y.J., Enriquez E., Peng X.F., Turng L.S. (2018). Highly stretchable and biocompatible strain sensors based on mussel−inspired super−adhesive self−healing hydrogels for human motion monitoring. ACS Appl. Mater. Interfaces.

